# Inflammation of the Pleura

**Published:** 1879-10

**Authors:** 


### INFLAMMATION OF THE PLEURA.

Inflammation of the pleura differs from that of the arachnoid, of which I spoke to you at our last meeting, from the fact that the exudation is never on the attached surface, but is found directly in the pleural sac itself. If it is serum it will be found first in the most dependent part of the sac. If plastic matter is effused it may be in the most dependent portion, but generally it remains on the membrane from which it exuded, and covers the whole surface. This causes the lung to adhere to the ribs. The respiratory force will cause the lung to move, but it goes with a jump. The organization going on, the adhesion will be more complete and the lung at the end of twelve or fourteen days is completely fastened to the ribs. One form of pleurisy results in the exudation of much lymph with some effusion of serum. This is acute pleurisy. When there is a moderate amount of lymph with a great quantity of serum, it is sub-acute pleurisy, hydrorax, or pleurisy proper. When there is pus in the pleural cavity as a product of inflammatory action it is called empyema. There is another form associated with a perforation of the lung and the introduction of air, which is called pneumothorax. In any case there is usually inflammation of the entire membrane.

*Acute Pleurisy.*—A very few times in your life will you meet this form of pleurisy. You will suddenly be called to a patient who has been seized with *intense pain* in his side, which has come on suddenly. If he has been sitting up he is pressing the affected side with his hand to prevent motion of the ribs as far as possible. If he is in bed he will be upon the inflamed side. As a rule pleurisy does not begin with a chill; countenance is pale; pulse is tense and frequent. But you cannot be sure of your diagnosis before you get the friction sound; for the first few hours you will not be able to get this sound, the fibrinous exudation not having had time to form or produce it. This sound is generally double, occurring both with expiration and inspiration, but it may be single. Laennec compares it to the sound produced by new leather. When this is heard the disease can scarcely be confounded with any other.

This disease, by common consent, is better controlled by the lancet than any other serious inflammation. Cups with scarification are to be applied to the affected side, these may be repeated two or three times after proper intervals.

When the pain has subsided, blisters may be applied, to overcome whatever inflammation remains. The treatment then must be decidedly antiphlogistic to prevent abundant effusion. There is no occasion for diuretics. Diaphoretics are made use of in the latter stages. Fomentations of warm water and the like may be applied to the affected side. Not much constitutional treatment required. This membrane, in cases which recover, is left and is organized. Contraction of side and shortness of breath will occur in every form of pleurisy. This does not take place until several weeks after the attack and continues for three or four years, during which time the membrane becomes absorbed.

*Sub-Acute Pleurisy.*—In this form there is a large amount of serous fluid with a moderate amount of lymph. The disease was formerly called hydrothorax. It is insidious in its attack and is attended with no pain unless it proceeds from the acute form. You will not probably see these cases until the disease has made considerable progress. The diagnosis will especially be distinct from phthisis from the protracted cough, paleness and emaciation. There will always be a cough which is almost dry, there being but very little expectoration. The character of the expectoration will be different from that of phthisis, in being tough and accompanied by frothy mucus. There is shortness of breath and poor appetite. The pulse is frequent—these are about all the rational signs. But we have the physical signs, which are diagnostic. The disease is almost always upon one side. The pleural cavity contains a *pool of water* and upon this are found the physical signs. First on inspection: There is a filling out of the intercostal spaces, especially at the lower part of the chest. The ribs are elevated and fixed, not moving in respiration. By measurement we ascertain that the affected side is the largest. There is absolute dullness on percussion as far up as the fluid extends. From the condensation of lung tissue the resonance will be less than natural. In making this examination the ear must be applied as far down as the ninth rib behind. Over that portion of the chest which contains water there will be heard no respiratory sound, and when the chest is *full* of water no sound can be heard except at the root of the lung. Absolute silence in respiration always denotes one of three things—watery effusion, a large tumor, or pneumonia with fibrinous bronchitis. When we have, as in pneumonia, hepatization, we will always get the respiratory sound which is tubular. This is a diagnostic mark between pneumonia and hydrothorax. When, in carrying my ear up to get the level of the water, I get the inspiratory and expiratory sound, I know I am over the lung, and that the sound is not a conducted one. Another mode of ascertaining the level of the fluid is by listening to the voice. Let the patient count, for this is better than talking. When we reach the level of the fluid, the voice sound which before was distinct and deep, will be full of resonance and in a low key. We shall also, in some cases, get a trembling sound, but upon this I do not rely. These, then, are the principal aids in the diagnosis, and when we get these there can be no doubt but that we have sub-acute pleurisy.

I suppose a lung was never so compressed as not to admit air into the bronchial tubes. This, fortunately, is one of the diseases which we may expect to cure, but not without proper treatment. For a year or two, and very often through life, we shall have deformity of the chest if the effusion has been of any amount, and the case has been at all protracted. This arises from the shrinkage of the affected side after removal of the fluid, upon the principle that "*Natura abhoruet vacuo,*" the adhesions preventing the complete restoration of the lung. We are also to notice the effect upon other organs. The heart is pushed to the opposite side of the fluid. The liver is crowded downwards, and once in a great while this organ is displaced.

Since the inflammatory nature of this trouble has been recognized, we have had great success in its management. While, when it was considered and treated as a mere dropsy. it was almost as fatal as phthisis. Being but a mild inflammatory action, it presents few terrors to the physician, but for its course it will require from two to four weeks, on an average. Very few cases prove fatal.

We are, on the one hand, to subdue the inflammation, and on the other to promote absorption of the fluid. Bleeding from the arm is unnecessary, but when we are called at an early stage, which is seldom the case, cups will be advisable, repeated as often as necessary. Over recent inflammations cups have great influence, while they do little good in subacute and chronic inflammations. As in other subacute inflammations, blisters are to be applied, and in doing this select three spots, one being placed on a new spot as the last has healed. Scarcely ever more than three are required. Now come active duretics, and among these I prefer potass. iod. gr. xxx. per day. If this does not subserve try potass. acet. et inf. digilatis ℨiij to ℥iv per day, or triplex diuretic pill, ℞ pulv. digitalis, pulv. sallæ mer., hyd. chlor. mit. aa grs. j. M. ft. pie noj cap. pil. ter in die, until the effect of the mercury is produced, then try potass. iod. again. In some cases mild counter-irritants, as in nervous women, but, as a rule, we are not to trust to these. Purgative and vapor baths sometimes are useful. This treatment will suffice in ordinary subacute pleurisy, but when all these measures fail we have but two resources, to do nothing or to use the trocar. Some physicians advise the early use of the trocar, but from fear of changing the serous effusion to pus, I do not like the practice. In some cases we are forced to use the trocar, but beforehand always use medical means, if the patient is not rapidly sinking.

*Empyema.*—Here we have the purulent matter instead of or with serum. It is not the grade of inflammation, but the constitution of the patient that gives character to this disease. The patient is apt to have chills and sweats, but we can not rely upon the symptoms, as they are deceitful. From the greater constitutional disturbance we shall be more apt to call this disease phthisis. Cough is worse with a great amount of constitutional disturbance and *expectoration.* Try the common means in use for this disease, viz., puncture of the chest.

Sometimes the pus has a tendency to point, from its being confined, as in an abcess. In these cases, as nature, though the best surgeon, is too slow, we are obliged to help her, in many cases hastening the evacuation of the fluid contents. Now and then, instead of an opening being made through the external intercostal spaces, the pus makes its escape through a perforation through the lung. This is a most unfavorable issue, and such patients usually die. These perforations, in most cases, depend upon a softening down of superficial tubercles. In all cases of empyema we must expect to puncture several times. The practice is to incise the skin, plunge in a trocar and canula, and after withdrawing these instruments, insert into the opening thus made a linen tent, which should be fastened by its free ends to the chest by means of adhesive strips. This tent may be removed every day or two to allow the pus to run out. But I am partial to Dr. Wyman's method, as practiced by Dr. Bowditch, of Boston. It consists in the use of the exhausting pump, and for this purpose I like the ordinary stomach pump. We are to use an extremely small trocar and a common exploring needle as sometimes made, is a good instrument for this purpose, or better still, the aspirator with a fine trocar. Before introducing the instrument it is well to *benumb* the part by firm pressure with the finger. In these cases it is not necessary to use the scalpel as we do when a large trocar is used. After the trocar has been withdrawn the canula may be pressed in some distance, as it will not hurt the lung if it touches it. Work the piston slowly. As the opening thus made is small it will close itself to the exclusion of air as soon as the canula is withdrawn. This last method is peculiarly desirable before we have ascertained that the effusion is purulent by a previous operation. When we know that the cavity contains pus I do not know which method is best. We are to continue to draw off fluid until oppression is felt at the sternum, and to guard against drawing off too much, for fear of making a vacuum too great for the comfort of the patient. When the operation is performed in front it must be between the sixth and seventh rib, to avoid wounding the diaphragm. On the side we should choose between the seventh and eighth rib, and in the back between the eighth and ninth rib. I think that iodine injections for the prevention of further effusion of pus are attended with more harm than good. If I were to use injections of any sort I should prefer simple warm water. Tonics are more or less serviceable, and this is about all that can be done in the way of treatment. Even under the best management we must expect half of these patients to die.

*Pneumothorax with Empyema.*—This is chiefly met with in persons having tubercles. The urgency of the symptoms will depend upon the degree of collapse of the lung. By applying the ear to the chest and requiring patient to count we hear a hollow, ringing, and cavernous sound, which is called amphoric respiration. This metallic ring results either from a dropping of a portion of the fluid or from the bursting of a bubble of air. It resembles the sound produced by dropping a pin into a wine glass. Succession sound is produced by shaking the fluid in a cavity which contains air, and hence cannot occur in sub acute pleurisy. It can only occur when air and fluid co-exist in the pleural cavity. The place where the *amphoric resonance* is heard with most distinctness will correspond to the situation of the perforation. Pus will only decompose when exposed to air or when in contact with dead bone which seems to act like a ferment. It is rare to get resolution of pus into gaseous matter when secluded from the air even out of the body. In pneumothorax the patient is seized with violent spells of coughing from irritation of the fluids in the pleural cavity. During these fits of coughing this is expelled and the patient feels relieved until the pus again reaches the perforation. Furthermore the patient will be compelled to lie in that position by which the opening will be out of reach of the fluid.

These cases are always desperate, for besides having two very fatal diseases (tubercles and empyema) associated with it, we have this last also. Our object should be to prolong life to the utmost limit by good food and stimulants. We may also apply with propriety some counter-irritant to the chest, as zinci iodid. Furthermore, as it is better for the pus to escape through the walls of the chest than through the lung, we are to make a permanent opening.—*Hospital Gazette.*

